# 961. Population Level Insights into Which Burn Survivors Receive Laser Therapy for Hypertrophic Scars

**DOI:** 10.1093/jbcr/irag033.539

**Published:** 2026-04-06

**Authors:** Chandler S Hinson, Allen Green, Clifford C Sheckter

**Affiliations:** Arnold Luterman Regional Burn Center, Huntsville, AL; Stanford University School of Medicine, Palo Alto, CA; Stanford University School of Medicine, Palo Alto, CA

## Abstract

**Introduction:**

Hypertrophic scarring, a frequent complication of burn injuries, leads to pain, functional impairment, and psychological distress. Laser therapy, particularly fractional ablative lasers and pulsed dye lasers, has become a standard treatment to improve scar symptoms and quality of life for burn survivors. From a population level, little is known regarding what types of burn survivors receive laser therapy and whether barriers exist.

**Methods:**

State Ambulatory Surgery and Services Databases (2014–2019) for New York and Florida were queried to identify patients receiving laser therapy for hypertrophic scars. State level ambulatory surgery databases were chosen given laser therapy is an outpatient procedure, and these databases cover all payers. Comparative analyses included demographic and socioeconomic factors, assessed vis-à-vis a national inpatient burn cohort from the 2020 Healthcare Cost and Utilization Project National Inpatient Sample. Logistic regression evaluated predictors of laser therapy utilization while Poisson regression evaluated temporal trends.

**Results:**

Among 709 laser therapy recipients, compared to the national inpatient burn cohort, these patients were significantly younger (mean age 28 vs 40, (95% beta coefficient: –0.001, 95% CI –0.001 to –0.002), more likely to be female (52.6% vs 33.7%, 95% aOR CI 1.87–2.56), more likely to be non-white (51.8% vs 42.7%, 95% aOR CI 1.23–1.69), more likely to be of the highest income quartile (24.5% vs 14.7%, aOR 95% CI 4.25; 95% CI 3.26–5.66), and more likely to reside in high-population areas (91.8% vs 72.5%). While 37.5% of all burn admissions are from the lowest income quartile, only 29.5% of all laser cases were low income (Fig. 1). In comparison, 24.5% of all laser cases were in the highest income quartile compared to 14.7% of all burn admissions. Insurance status was broadly similar across groups, though regression analysis suggested that private insurance conferred a modestly higher likelihood of receiving laser therapy (aOR 1.24; 95% CI 1.05–1.45). There was a significant increase in the incidence of laser treatments over the study period (IRR [95%]: 1.29 [1.23–1.35]) (Fig. 2).

**Conclusions:**

Recipients of laser therapy for hypertrophic scars differ demographically from the US inpatient burn population. Notably laser recipients are younger, wealthier, more non-white, and reside in urban/suburban population centers. While some of these variables may be related to underlying differences in which patients form hypertrophic scars, differential access to laser therapy likely prohibits some burn survivors from receiving treatment.

**Applicability of Research to Practice:**

Burn centers should be aware of disparities in laser access and evaluate opportunities to engage burn survivors who live in rural areas or come from low-income backgrounds to receive laser therapy for hypertrophic scars.

**Funding for the study:**

N/A.

